# Performance evaluation of existing immunoassays for *Clonorchis sinensis* infection in China

**DOI:** 10.1186/s13071-018-2612-3

**Published:** 2018-01-15

**Authors:** Hong-Mei Li, Men-Bao Qian, Yi-Chao Yang, Zhi-Hua Jiang, Kang Wei, Jia-Xu Chen, Jun-Hu Chen, Ying-Dan Chen, Xiao-Nong Zhou

**Affiliations:** 1 National Institute of Parasitic Diseases, Chinese Center for Disease Control and Prevention, Shanghai, People’s Republic of China; 2 WHO Collaborating Center for Tropical Diseases, Shanghai, People’s Republic of China; 3 National Center for International Research on Tropical Diseases, Shanghai, People’s Republic of China; 4 Key Laboratory of Parasite and Vector Biology, Ministry of Health, Shanghai, People’s Republic of China; 5 Guangxi Center for Disease Control and Prevention, Nanning, Guangxi People’s Republic of China; 6 Hengxian Center for Disease Control and Prevention, Hengxian, Nanning, Guangxi People’s Republic of China

**Keywords:** Clonorchiasis, *Clonorchis sinensis*, Immunodiagnosis, Evaluation, Sensitivity, Specificity, Youden’s index

## Abstract

**Background:**

Clonorchiasis ranks among the most important food-borne parasitic diseases in China. However, due to low compliance to traditional fecal examination techniques in the general population and medical personnel, immunodiagnosis is expected. This study evaluated, in parallel, the performance of four immunodiagnostic kits detecting clonorchiasis in China.

**Results:**

A bank with 475 sera was established in this study. Except for the low performance of the kit detecting IgM, the other three kits detecting IgG showed sensitivities ranging from 81.51% (194/238) to 99.16% (236/238). Higher sensitivity was presented in heavy infection intensity [89.47% (68/76) to 100% (76/76)]. Among the four kits, the overall specificity varied from 73.42% (174/237) to 87.34% (207/237). It was observed that the specificity was lower in the sera of the participants living in clonorchiasis-endemic areas but without any parasite infection [67.5% (81/120) to 90% (108/120)], as compared to those from the non-endemic area [94% (47/50) to 98% (49/50)]. The cross-reaction rate varied from 14.93% (10/67) to 31.34% (21/67). Youden’s index was -0.022, 0.689, 0.726, and 0.802 for kits T1, T2, T3 and T4, respectively. Repeatability was high in all four kits.

**Conclusions:**

Three immunodiagnosis kits targeting IgG antibody had high performance on detecting chronic *Clonorchis sinensis* infection, but that detecting IgM antibody had not. The kits detecting IgG antibody also showed high sensitivity in heavy infection intensity. Research on immunological diagnosis of clonorchiasis is expected to be strengthened to improve the sensitivity in light infection and specificity.

**Electronic supplementary material:**

The online version of this article (10.1186/s13071-018-2612-3) contains supplementary material, which is available to authorized users.

## Background

Clonorchiasis is caused by an infection with *Clonorchis sinensis* (Opisthorchiidae), through the consumption of raw or undercooked freshwater fish, and ranks among the most important food-borne parasitic diseases in public health [1–3]. Clonorchiasis is highly endemic in China, Republic of Korea, Vietnam and parts of Russia [4–6]. A total of 15 million people are estimated to be infected worldwide, 13 million of which live in China [6, 7]. Adult worms inhabit intrahepatic bile ducts, which can lead to liver and biliary diseases, including cholangitis, cholelithiasis, cholecystitis and cholangiocarcinoma [5, 8]. *Clonorchis sinensis* was re-classified as a group 1 carcinogen by the International Agency for Research on Cancer (IARC) in 2009 [9, 10]. Nearly 5000 new cholangiocarcinoma cases attributed to *C. sinensis* infection occur annually [6, 7].

Accurate and rapid diagnosis of *C. sinensis* infection is crucial for early case management and treatment; traditional fecal examination for *C. sinensis* eggs is the “gold standard” for diagnosis. Among those fecal examination methods, the Kato-Katz method is most widely applied, as it is simple, inexpensive, and quantitative [11–13]. The water washing precipitation technique and formalin-ether concentration technique (FECT) are other frequently used techniques [11, 14]. However, there exist obvious limitations. First, the sensitivity of traditional techniques is low, especially in low-burden infections [15]. Secondly, it is becoming more difficult to collect stool samples due to the increasing low-compliance of general population. Furthermore, medical personnel are also not reluctant to deal with fecal matter. In spite of molecular techniques including polymerase chain reaction (PCR) and loop-mediated isothermal amplification (LAMP) being successfully applied in the diagnosis of *C. sinensis* infection [16–19], sample contamination and high costs hinder their application in large-scale surveys in the field.

Up to date immunological technology is an important domain of research in the field of clonorchiasis in China, e.g. enzyme-linked immunosorbent assay (ELISA) and its new derivate, the gold immunochromatographic assay (GICA), which is expected to play more roles in field. The purpose of this study was to evaluate, in parallel, the performance of available immunodiagnostic kits detecting clonorchiasis in China, which has not yet been evaluated systematically.

## Methods

### Selection of immunodiagnostic tests

Only once a product met following requirements, could it be enrolled in this study. First, the specimen applied in the test is sera. Secondly, the test must be produced formally by a company in China.

Four immunoassay kits were enrolled. Three kits were indirect-ELISA kits (named T1, T2 and T3) and the other a GICA kit (named T4). T1 detected IgM antibody, whereas T2, T3, and T4 detected IgG antibody. The antigen of T1 and T2 was the crude soluble adult worm’s extract, and the antigen of T3 and T4 was recombinant antigen from yeast. Brief introductions of the four kits are provided in Table 1.

**Table 1:** Brief introduction of the four kits tested

Kit	Kit name	Assay type	Antibody type	Antigen	Source	Time required per run	Sample volume (μl)	Extra supplies^a^
T1	Test kit for IgM to *Clonorchis sinensis* (ELISA)	Indirect ELISA	IgM	Crude antigen	Adult worms	2 h	5	Yes
T2	Test kit for IgG to *Clonorchis sinensis* (ELISA)	Indirect ELISA	IgG	Crude antigen	Adult worms	1.5 h	50	Yes
T3	Detection kit for IgG antibody to *Clonorchis sinensis* (ELISA)	Indirect ELISA	IgG	Recombinant antigen	Yeast	1.5 h	10	Yes
T4	Gold Immunochromatography assay for IgG antibody to *Clonorchis sinensis*	GICA	IgG	Recombinant antigen	Yeast	10 min	9	No

^a^Required additional equipment to finish the test, such as incubator and microplate reader

### Sera bank

The sera bank applied in this study included four types of sera, namely the sera from patients with mere *C. sinensis* infection, from people living in the clonorchiasis endemic area and without any parasite infection (control 1), from people living in the non-clonorchiasis endemic area and without any parasite infection (control 2), and from cases infected with parasites other than *C. sinensis* (control 3).

### Sera from endemic area

The sera from those infected with *C. sinensis* and control 1 were collected through a cross-sectional survey in Hengxian county, Guangxi Zhuang Autonomous Region, China, where *C. sinensis* infection is highly endemic [20]. One stool sample was collected from each participant. Then, both the Kato-Katz method and the water washing precipitation method were applied. In the Kato-Katz method, three smears with 41.7 mg feces in each one were prepared, which can be used to quantitate the eggs. Only qualitative results were presented in the water washing precipitation method. Only those without any parasite infection in three Kato-Katz smears and water washing precipitation method were enrolled as control sera. Only those with mere *C. sinensis* infection and without other parasites were enrolled as positive sera. For quantitation, those positive in the water washing precipitation method but negative in the Kato-Katz method were also excluded.

### Sera from non-endemic area and other parasitic diseases

Sera from the control group 2 were collected from healthy individuals living in Shanghai, where *C. sinensis* infection is not endemic. Sera from the control group 3 included those with schistosomasis, paragonimiasis, trichinellosis and soil-transmitted helminthiases (ascariasis, trichuriasis and hookworm disease). Sera from both control groups 2 and 3 were supplied by the sample preservation center in National Institute of Parasitic Diseases, Chinese Center for Disease Control and Prevention.

### Procedures

Sera were preserved at -80 °C and were thawed and agitated before use. Then, operation procedures were conducted according to the instruction manual of each kit. To analyze the repeatability, 45 sera samples sampled at random were used for second-round test.

### Statistical analysis

All data were analyzed using the SPSS software (Version 20.0, IBM Corp., New York, USA). *Clonorchis sinensis* infection was classified into three categories according to infection intensity through the Kato-Katz method, expressed by the eggs per gram of feces (epg): light infection (1–999 epg), moderate infection (1000–9999 epg) and heavy infection (≥ 10,000 epg). Based on the “gold standard” of fecal examination (combined the Kato-Katz method and water washing precipitation method), the sensitivity and specificity of four immunodiagnostic tests were calculated, and the 95% confidence intervals (95% CI) were also presented by normal approximation method. Sensitivity is the number of true positives / (number of true positives + number of false negativies), and 95% CI of sensitivity = sensitivity ± 1.96*SQRT(sensitivity*(1 – sensitivity)/238. Specificity is the number of true negatives / (number of true negatives + number of false positives), and 95% CI of specificity = specificity ± 1.96*SQRT(specificity *(1 – specificity)/237. McNemar’s test was used to compare the sensitivity and specificity between each group of two kits, in which Fisher’s exact test was applied to yield a *P*-value to assess the difference between each group of two kits. *P*-values < 0.5 indicate that the difference was statistically significant between those two kits. Youden’s index was expressed as the sum of sensitivities and specificities subtracted by one [21], which represented the difference between the true-positive rate and the false-positive rate, and is widely used in evaluating the accuracy and performance of diagnostic tests. Youden’s index varies between -1 and 1, whereas the index < 0 indicates that the kit is meaningless in practice. The higher the Youden’s index is, the more accurate the diagnostic performance is [22].

## Results

### Characteristics of the sera bank

The sera bank in this study included 475 sera. Among them, 238 cases with *C. sinensis* infection were enrolled, consisting of 81 with light infection, 81 with moderate infection, and 76 with heavy infection. The bank included 120 negative sera from the clonorchiasis endemic area and another 50 negative sera from the non-clonorchiasis endemic area. Additionally, 67 sera were from cases with other parasitic diseases, including 20 with schistosomiasis, 10 with paragonimiasis, 10 with trichinellosis, 9 with ascariasis, 9 with trichuriasis and 9 with hookworm disease.

### Sensitivity

The total sensitivities of the four test kits (T1, T2, T3 and T4) are shown in Table 2. There was a markedly low sensitivity for one kit (T1), whereas the other three (T2, T3 and T4) were high, and the difference was significant in all two-by-two kit comparisons (McNemar’s tests: T1 vs T2: *P* < 0.0001; T1 vs T3: *P* < 0.0001; T1 vs T4: *P* < 0.0001; T2 vs T3: *P* < 0.0001; T2 vs T4: *P* < 0.0001; T3 vs T4: *P* = 0.006) (Table 3). In the light infection groups, T1 still presented a markedly low sensitivity, while T3 was outstanding high (97.53%), and significant differences were also detected in all pairwise comparisons between all kits in the light infection group (McNemar’s test: T1 vs T2: *P* < 0.0001; T1 vs T3: *P* < 0.0001; T1 vs T4: *P* < 0.0001; T2 vs T3: *P* < 0.0001; T2 vs T4: *P* < 0.0001; T3 vs T4: *P* = 0.021) (Table 3). In the moderate and heavy infection groups, T1 remained the same low sensitivity. However, outstanding high sensitivities were found in the other three kits (T2, T3 and T4). More remarkable, partial sensitivities were reached 100% (e.g. T3 in both moderate and heavy infection group, and T4 in heavy infection group).

**Table 2:** Sensitivity of four immunodiagnostic kits for clonorchiasis in China

Kit	Light infection (*n* = 81)		Moderate infection (*n* = 81)		Heavy infection (*n* = 76)		Total (*n* = 238)	
	No. of positive	Sensitivity (%) (95% CI)	No. of positive	Sensitivity (%) (95% CI)	No. of positive	Sensitivity (%) (95% CI)	No. of positive	Sensitivity (%) (95% CI)
T1	13	16.05 (11.39–18.43)	12	14.81 (10.3–19.33)	8	10.53 (6.63–14.43)	33	13.87 (9.47–18.26)
T2	51	62.96 (56.83–66.09)	75	92.59 (89.27–95.92)	68	89.47 (85.57–93.37)	194	81.51 (76.58–86.44)
T3	79	97.53 (95.56–98.54)	81	100 (100)	76	100 (100)	236	99.16 (98.00–100)
T4	71	87.65 (83.47–89.79)	79	97.53 (95.56–99.50)	76	100 (100)	226	94.96 (92.18–97.74)

**Table 3:** Comparison of sensitivity between kits by McNemar’s test

Kit	T1	T2	T3
Total sera (*n* = 238)			
T1			
T2	< 0.0001*		
T3	< 0.0001*	< 0.0001*	
T4	< 0.0001*	< 0.0001*	0.006*
Light infection intensity			
T1			
T2	< 0.0001*		
T3	< 0.0001*	< 0.0001*	
T4	< 0.0001*	< 0.0001*	0.021*
Moderate infection intensity			
T1			
T2	< 0.0001*		
T3	ns	ns	
T4	< 0.0001*	0.289	ns
Heavy infection intensity			
T1			
T2	< 0.0001*		
T3	ns	ns	
T4	ns	ns	ns

*Abbreviation*: *ns* no statistics

*P < 0.05

### Specificity

Table 4 shows the specificity data for the four kits. Among the overall sera, T3 kit showed significantly lower specificity than that in the other three kits. McNemar’s tests demonstrated that there existed significant differences between T3 kit and the other three kits (T1 vs T3: *P* = 0.003; T2 vs T3: *P* < 0.0001; T3 vs T4: *P* = 0.001) (Table 5). The specificity was lower in sera from the endemic area (67.5–90%) compared to that from the non-endemic area (94–98%) (Table 4). Significant differences were found when testing healthy sera from endemic area between T3 kit and the other three kits, as well as T2 kit and T4 kit (McNemar’s test: T1 vs T3: *P* < 0.0001; T2 vs T3: *P* < 0.0001; T2 vs T4: *P* = 0.035; T3 vs T4: *P* = 0.023) (Table 5). However, the difference was not significant in detecting those from the non-endemic area between the four kits (Table 5). The cross-reaction rate with other heterologous sera varied between 14.93–31.34%, and high cross-reaction was presented in paragonimosis (70%, 28/40) (Table 4 and Additional file 1: Table S1). The difference was significant when the T4 kit was compared to the T1 kit and the T3 kit (McNemar’s test: T1 vs T4: *P* = 0.031; T3 vs T4: *P* = 0.035) (Table 5).

**Table 4:** Specificity of four immunodiagnostic kits for clonorchiasis in China

Kit	Negative sera (Control 1) (*n* = 120)		Healthy sera (Control 2) (*n* = 50)		Heterologous sera (Control 3) (*n* = 67)		Total (*n* = 237)	
	No. of false positives	Specificity (%) (95% CI)	No. of false positives	Specificity (%) (95% CI)	No. of false positives	Specificity (%) (95% CI)	No. of false positives	Specificity (%) (95% CI)
T1	16	86.67 (80.58–92.75)	2	96 (90.57–100)	20	70.15 (59.68–81.5)	38	83.97 (79.29–88.64)
T2	12	90 (84.63–95.37)	1	98 (94.12–100)	17	74.63 (64.63–85.37)	30	87.34 (83.11–91.58)
T3	39	67.5 (59.10–75.88)	3	94 (87.42–100)	21	68.66 (58.05–80.18)	63	73.42 (67.79–79.04)
T4	23	80.83 (73.79–87.88)	2	96 (90.57–100)	10	85.07 (76.81–93.77)	35	85.23 (80.72–89.75)

**Table 5:** Comparison of specificity between kits by McNemar’s test

Kit	T1	T2	T3
Total sera (*n* = 237)			
T1			
T2	0.256		
T3	0.003*	< 0.0001*	
T4	0.766	0.542	0.001*
Negative sera from endemic areas (Control 1)			
T1			
T2	0.503		
T3	< 0.0001*	< 0.0001*	
T4	0.210	0.035*	0.023*
Healthy sera from non-endemic areas (Control 2)			
T1			
T2	1		
T3	1	0.625	
T4	1	1	1
Heterologous sera (Control 3)			
T1			
T2	0.629		
T3	1	0.481	
T4	0.031*	0.143	0.035*

*P < 0.05

### Accuracy

**Fig. 1:**
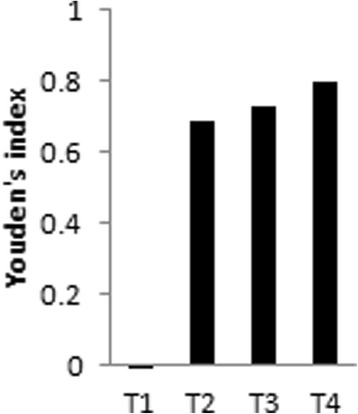
Youden’s index for the four kits

Youden’s indices for the four kits (T1, T2, T3 and T4) were -0.022, 0.689, 0.726 and 0.802, respectively (Fig. 1). Thus, T4, T3 and T2 kits showed better accuracy, especially T4, whereas T1 demonstrated low value according to the Youden’s index.

### Repeatability

The repeatability rates in the four kits (T1, T2, T3 and T4) were 92.86%, 95.35%, 95.35% and 95.35%, respectively.

## Discussion

Clonorchiasis is becoming the most important food-borne parasitic diseases in China, due to its high disease burden caused by high prevalence as well as heavy infection intensity. Because of the raw-fish-eating habits rooted in the culture and the chronicity of this infection, people in endemic areas do not realize the danger of clonorchiasis. Thus, the prevalence of clonorchiasis is still increasing in China, compared to the significant decrease and even elimination of other parasitic diseases such as soil-transmitted helminthiases, schistosomiasis, malaria and filariasis [23–26]. Chemotherapy has been demonstrated to be the most effective choice to control clonorchiasis in the short-term [2, 27]. Thus, identifying those who need chemotherapy is important.

Compared to traditional fecal examination or newly developed molecular technology, immunodiagnosis is an accepted method due to its low cost, high speed and suitability in large studies. In this study, we systematically evaluated four immunodiagnostic kits developed in China using a sera bank under well-controlled conditions. Compared to those detecting IgG antibody (T2, T3, and T4), the kit detecting IgM antibody (T1) demonstrated poor performance. IgM antibody appears early after infection with *C. sinensis* and exists for only a few weeks [28, 29]. IgM antibody has been commonly used as the index of early diagnosis of new infection with parasitic diseases (e.g. schistosomiasis) [30, 31]. The appearance of IgG antibody follows IgM and lasts for a long period [28, 29]. The sera infected with *C. sinensis* in this study were from a high-endemic area [20]. This study showed that immunodiagnosis for IgM antibody is not an adequate test for those from a high endemic area of clonorchiasis.

The performance of three other diagnostic kits (T2, T3, and T4) detecting IgG antibody was relatively high. It was also demonstrated that higher sensitivity was presented in heavier infection intensity samples, and the highest number of false-negative results was present in the light-infected serum samples, which is consistent to the difference in immune reaction. The sensitivity was significantly different between any two kits in detecting light infection intensity. Although high specificity (≥ 94%) was presented in those from the non-clonorchiasis endemic area, the specificity was lower in those from the endemic area (≤ 90%). This may be explained by a potential past infection with *C. sinensis*. The specificities of three kits detecting IgG were also influenced by cross-reactivity to the heterologous sera with paragonimiasis and schistosomiasis. These results suggest that diagnosis should be carefully made in people who come from the areas that are co-endemic with clonorchiasis and paragonimiasis or schistosomiasis. In addition to high sensitivity and specificity, the key characteristics of diagnostic kits also include other parameters [32]. Repeatability should be paid particular attention. The four kits in this study showed good repeatability and stability.

There were limitations of these kits, however, that should be improved. First, sensitivity is inadequate in light infection intensity, which is an important performance requirement, both for detecting infection in low endemic areas and during the treatment period. Indeed, the infection intensity in the light infection group in this study was relatively high (mean epg of 393.28). Thus, their performance for lower infection intensity should be evaluated. Secondly, the specificity needs to be improved, particularly the low performance in distinguishing the healthy in endemic areas. Thirdly, paragonimosis showed the strongest cross-reaction, which will limit the test’s use in paragonimosis-endemic areas.

## Conclusions

The performance of three immunodiagnosis kits detecting IgG antibody was relatively high in detecting clonorchiasis infection, but that detecting IgM antibody had relatively low performance. Those detecting IgG antibody had high sensitivity for heavier infection intensities, but their performance in lower infection intensity and specificity should be improved. The T3 kit, with the highest sensitivity of the kits in both heavier infection intensity sera and light infected ones, may be expected to detect more positive sera in light endemic areas. However, its specificity needs to be improved. Although the specificity of T4 is relatively higher than T3 kit, its sensitivity in light infection intensity needs to be improved. However, simplicity and quickness are important characteristics of the T4 kit.

## Additional file

Additional file 1: Table S1. Specificity of four immunodiagnostic kits for clonorchiasis in China. (DOCX 33 kb)

## Abbreviations

95% CI: 95% confidence interval
ELISA: Enzyme-linked immunosorbent assay
epg: eggs per gram of feces
FECT: Formalin-ether concentration technique
GICA: The gold immunochromatographic assay
IARC: International Agency for Research on Cancer
LAMP: Loop-mediated isothermal amplification
PCR: Polymerase chain reaction

## References

[1] Sripa B, Kaewkes S, Intapan PM, Maleewong W, Brindley PJ (2010). Food-borne trematodiases in Southeast Asia epidemiology, pathology, clinical manifestation and control. Adv Parasitol.

[2] Lun ZR, Gasser RB, Lai DH, Li AX, Zhu XQ, XB Y, Fang YY (2005). Clonorchiasis: a key foodborne zoonosis in China. Lancet Infect Dis.

[3] Qian MB, Utzinger J, Keiser J, Zhou XN (2016). Clonorchiasis. Lancet.

[4] Keiser J, Utzinger J (2009). Food-borne trematodiases. Clin Microbiol Rev.

[5] Hong ST, Fang Y (2012). *Clonorchis sinensis* and clonorchiasis, an update. Parasitol Int.

[6] Qian MB, Chen YD, Liang S, Yang GJ, Zhou XN (2012). The global epidemiology of clonorchiasis and its relation with cholangiocarcinoma. Infect Dis Poverty.

[7] Furst T, Keiser J, Utzinger J (2012). Global burden of human food-borne trematodiasis: a systematic review and meta-analysis. Lancet Infect Dis.

[8] Shin HR, JK O, Masuyer E, Curado MP, Bouvard V, Fang YY (2010). Epidemiology of cholangiocarcinoma: an update focusing on risk factors. Cancer Sci.

[9] International Agency for Research on Cancer. A review of human carcinogens, Part B: Biological agents. International Agency for Research on Cancer. 2012;100:1–30.

[10] Bouvard V, Baan R, Straif K, Grosse Y, Secretan B, El Ghissassi F, et al. A review of human carcinogens, Part B: Biological agents. Lancet Oncol. 2009;10:321–2. 10.1016/s1470-2045(09)70096-8 19350698

[11] Qian MB, Yap P, Yang YC, Liang H, Jiang ZH, Li W (2013). Accuracy of the Kato-Katz method and formalin-ether concentration technique for the diagnosis of *Clonorchis sinensis*, and implication for assessing drug efficacy. Parasit Vectors.

[12] Hong ST, Choi MH, Kim CH, Chung BS, Ji Z (2003). The Kato-Katz method is reliable for diagnosis of *Clonorchis sinensis* infection. Diagn Microbiol Infect Dis.

[13] Choi MH, Ge T, Yuan S, Hong ST (2005). Correlation of egg counts of *Clonorchis sinensis* by three methods of fecal examination. Korean J Parasitol.

[14] Pan L, Chen C, Zhang DF. Comparison of four laboratory detection methods of clonorchiasis. Parasit Infect Dis. 2012;10:233–4.

[15] Han S, Zhang X, Wen J, Li Y, Shu J, Ling H, Zhang FA (2012). Combination of the Kato-Katz methods and ELISA to improve the diagnosis of clonorchiasis in an endemic area, China. PLoS One.

[16] Cai XQ, Yu HQ, Li R, Yue QY, Liu GH, Bai JS (2014). Rapid detection and differentiation of *Clonorchis sinensis* and *Opisthorchis viverrini* using real-time PCR and high resolution melting analysis. Sci World J.

[17] Sanpool O, Intapan PM, Thanchomnang T, Janwan P, Lulitanond V, Doanh PN (2012). Rapid detection and differentiation of *Clonorchis sinensis* and *Opisthorchis viverrini* eggs in human fecal samples using a duplex real-time fluorescence resonance energy transfer PCR and melting curve analysis. Parasitol Res.

[18] Rahman SMM, Song HB, Jin Y, JK O, Lim MK, Hong ST, Choi MH (2017). Application of a loop-mediated isothermal amplification (LAMP) assay targeting cox1 gene for the detection of *Clonorchis sinensis* in human fecal samples. PLoS Negl Trop Dis.

[19] Kaewkong W, Intapan PM, Sanpool O, Janwan P, Thanchomnang T, Laummaunwai P (2013). Molecular differentiation of *Opisthorchis viverrini* and *Clonorchis sinensis* eggs by multiplex real-time PCR with high resolution melting analysis. Korean J Parasitol..

[20] Qian MB, Chen YD, Yang YC, Lu MF, Jiang ZH, Wei K (2014). Increasing prevalence and intensity of foodborne clonorchiasis, Hengxian County, China, 1989–2011. Emerg Infect Dis.

[21] Xu J, Peeling RW, Chen JX, Wu XH, Wu ZD, Wang SP (2011). Evaluation of immunoassays for the diagnosis of *Schistosoma japonicum* infection using archived sera. PLoS Negl Trop Dis.

[22] Chen F, Xue Y, Tan MT, Chen P (2015). Efficient statistical tests to compare Youden index: accounting for contingency correlation. Stat Med.

[23] Sudomo M, Chayabejara S, Duong S, Hernandez L, Wu WP, Bergquist R. Elimination of lymphatic filariasis in Southeast Asia. Adv Parasitol. 2010;72:205–33. 10.1016/S0065-308X(10)72008-X 20624533

[24] Coordinating Office of the National Survey on the Important Human Parasitic Diseases. A national survey on current status of the important parasitic diseases in human population. Zhongguo Ji Sheng Chong Xue Yu Ji Sheng Chong Bing Za Zhi. 2005;23(Suppl. 5):332–40. 16562464

[25] Zhou XN, Guo JG, XH W, Jiang QW, Zheng J, Dang H (2007). Epidemiology of schistosomiasis in the People's Republic of China, 2004. Emerg Infect Dis.

[26] Feng J, Xiao H, Xia Z, Zhang L, Xiao N. Analysis of malaria epidemiological characteristics in the People's Republic of China, 2004–2013. Am J Trop Med Hyg. 2015;93:293–9. 10.4269/ajtmh.14-0733 PMC4530750 26078326

[27] Choi MH, Park SK, Li Z, Ji Z, Yu G, Feng Z (2010). Effect of control strategies on prevalence, incidence and re-infection of clonorchiasis in endemic areas of China. PLoS Negl Trop Dis.

[28] Quan FS, Matsumoto T, Shin YO, Min YK, Yang HM, Othman T, Lee JB (2004). Relationships between IgG, IgM, IgE and resistance to reinfection during the early phase of infection with *Clonorchis sinensis* in rats. Immunol Investig.

[29] Chen CY, Shin JW, Chen SN, Hsieh WC (1989). A Preliminary study of clinical staging in clonorchiasis. Zhongguo Ji Sheng Chong Xue Yu Ji Sheng Chong Bing Za Zhi..

[30] Espirito-Santo MC, Sanchez MC, Sanchez AR, Alvarado-Mora MV, Castilho VL, Goncalves EM (2014). Evaluation of the sensitivity of IgG and IgM ELISA in detecting *Schistosoma mansoni* infections in a low endemicity setting. Eur J Clin Microbiol Infect Dis.

[31] Tang Y, Wang Y, Shi XH, Xu WM, Gan XX (2009). The dynamic observations on the development of IgG/IgM antibodies before and after treatment of rabbit infection with *Schistosoma japonicum* using different kinds of schistosomal antigens. Zhongguo ren shou gong huan bing za zhi.

[32] Johansen MV, Sithithaworn P, Bergquist R, Utzinger J (2010). Towards improved diagnosis of zoonotic trematode infections in Southeast Asia. Adv Parasitol.

