# Supplementary material for: Performance evaluation of existing immunoassays for Clonorchis sinensis infection in China
Source: Parasit Vectors. 2018 Jan 15;11:35. doi: 10.1186/s13071-018-2612-3 (PMC5769360; doi:10.1186/s13071-018-2612-3)
Supplement: Additional file 1: Table S1. — Specificity of four immunodiagnostic kits for clonorchiasis in China. (DOCX 33 kb) [file 13071_2018_2612_MOESM1_ESM.docx]

**Additional file 1: Table S1.** Specificity of four immunodiagnostic kits for clonorchiasis in China

| **Kit** | **No. of false positives** | | | | | | | | | **Specificity (%) (95% CI)** | | | |
| --- | --- | --- | --- | --- | --- | --- | --- | --- | --- | --- | --- | --- | --- |
|  | **Negative sera (Control 1) (*n* = 120)** | **Healthy sera (Control 2) (*n* = 50)** | **Schistosomiasis (*n* = 20)** | **Paragonimiasis (*n* = 10)** | **Trichinellosis (10)** | **Ascariasis (*n* = 9)** | **Hookworm disease (*n* = 9)** | **Trichuriasis (*n* = 9)** | **Total (*n* = 237)** | **Negative sera (Control 1) (*n* = 120)** | **Healthy sera (Control 2) (*n* = 50)** | **Heterologous sera (Control 3) (*n* = 67)** | **Total (*n* = 237)** |
| T1 | 16 | 2 | 3 | 8 | 0 | 4 | 1 | 4 | 38 | 86.67 (80.58–92.75) | 96（90.57–100） | 70.15（59.68–81.5） | 83.97（79.29–88.64） |
| T2 | 12 | 1 | 8 | 8 | 0 | 0 | 0 | 1 | 30 | 90（84.63–95.37） | 98（94.12–100） | 74.63（64.63–85.37） | 87.34（83.11–91.58） |
| T3 | 39 | 3 | 7 | 9 | 0 | 2 | 2 | 1 | 63 | 67.5（59.10–75.88） | 94（87.42–100） | 68.66（58.05–80.18） | 73.42（67.79–79.04） |
| T4 | 23 | 2 | 2 | 3 | 0 | 1 | 1 | 3 | 35 | 80.83（73.79–87.88） | 96（90.57–100） | 85.07（76.81–93.77） | 85.23（80.72–89.75） |
